# REDD1 is a determinant of low-dose metronomic doxorubicin-elicited endothelial cell dysfunction through downregulation of VEGFR-2/3 expression

**DOI:** 10.1038/s12276-021-00690-z

**Published:** 2021-10-25

**Authors:** Minsik Park, Joohwan Kim, Taesam Kim, Suji Kim, Wonjin Park, Kwon-Soo Ha, Sung Hwan Cho, Moo-Ho Won, Jeong-Hyung Lee, Young-Guen Kwon, Young-Myeong Kim

**Affiliations:** 1 Department of Molecular and Cellular Biochemistry, Kangwon National University School of Medicine, Chuncheon, Gangwon-do 24341 Republic of Korea; 2 Kangwon Institute of Inclusive Technology, Kangwon National University, Chuncheon, Gangwon-do 24341 Republic of Korea; 3 Department of Neurobiology, Kangwon National University School of Medicine, Chuncheon, Gangwon-do 24341 Republic of Korea; 4 Department of Biochemistry, Kangwon National University, Chuncheon, Gangwon-Do 24341 Republic of Korea; 5 Department of Biochemistry, College of Life Science and Biotechnology, Yonsei University, Seoul, 03722 Republic of Korea

**Keywords:** Biochemistry, Cell biology

## Abstract

Low-dose metronomic chemotherapy (LDMC) inhibits tumor angiogenesis and growth by targeting tumor-associated endothelial cells, but the molecular mechanism has not been fully elucidated. Here, we examined the functional role of regulated in development and DNA damage responses 1 (REDD1), an inhibitor of mammalian target of rapamycin complex 1 (mTORC1), in LDMC-mediated endothelial cell dysfunction. Low-dose doxorubicin (DOX) treatment induced REDD1 expression in cultured vascular and lymphatic endothelial cells and subsequently repressed the mRNA expression of mTORC1-dependent translation of vascular endothelial growth factor receptor (*Vegfr*)-*2/3*, resulting in the inhibition of VEGF-mediated angiogenesis and lymphangiogenesis. These regulatory effects of DOX-induced REDD1 expression were additionally confirmed by loss- and gain-of-function studies. Furthermore, LDMC with DOX significantly suppressed tumor angiogenesis, lymphangiogenesis, vascular permeability, growth, and metastasis in B16 melanoma-bearing wild-type but not *Redd1*-deficient mice. Altogether, our findings indicate that REDD1 is a crucial determinant of LDMC-mediated functional dysregulation of tumor vascular and lymphatic endothelial cells by translational repression of *Vegfr-2/3* transcripts, supporting the potential therapeutic properties of REDD1 in highly progressive or metastatic tumors.

## Cancer: Stress protein implicated in effects of low-dose metronomic chemotherapy

Sustained low-dose metronomic chemotherapy impairs the function of tumor-associated blood vessels and lymphatic tissues due to the action of a stress response protein called REDD1. A research team from South Korea led by Young-Myeong Kim of Kangwon National University School of Medicine in Chuncheon showed that low doses of doxorubicin, a widely used anti-cancer drug, induces the expression of REDD1. This in turn inhibits the activity of vascular endothelial growth factor receptors-2/-3 that are needed for the formation of blood and lymphatic vessels, both of which can promote tumor growth. Cancer-bearing mice that lacked a working version of REDD1 did not respond to continuous low-dose treatment with doxorubicin, demonstrating the critical nature of this protein in mediating the drug regimen’s effects. The authors propose that pharmacologically enhancing REDD1 activity could help combat highly aggressive or metastatic tumors.

## Introduction

Growing solid tumors have a hypoxic environment due to an imbalance between oxygen supply and consumption, which in turn stimulates hypoxia-dependent expression of angiogenic and lymphangiogenic genes, including vascular endothelial growth factor (VEGF)^1^. As such, solid tumor growth is typically accompanied by angiogenesis and lymphangiogenesis via stimulation of VEGF receptor-1 (VEGFR-1), VEGFR-2, and VEGFR-3 on tumor-associated endothelial cells (TECs), subsequently improving energy metabolism, proliferation, and metastasis of tumor cells^2^. Thus, antiangiogenic and antilymphangiogenic strategies that block the VEGF/VEGFR pathway have been developed to treat several solid tumors in the clinic^1,2^.

Conventional chemotherapy at the maximal tolerated dose (MTD) results in impressive therapeutic responses against tumor cells^3^ but can also cause severe adverse effects, such vomiting, hair loss, cardiotoxicity, and drug resistance^3^. Thus, low-dose metronomic chemotherapy (LDMC) has been developed as a promising alternative strategy to avoid or minimize the side effects of MTD chemotherapy. The therapeutic effects of LDMC are associated with an increased sensitivity of activated TECs compared with other types of normal cells and tumor cells^3–5^. Thus, LDMC is considered an effective salvage treatment for patients with various malignancies^6^, possibly by attenuating tumor angiogenesis and lymphangiogenesis via functional dysregulation of TECs^6–9^.

Several molecular mechanisms underlying LDMC efficacy, including direct inhibition of the growth of TECs but not tumor cells, have been proposed^4,5^. The predominant modes of action are thought to involve inhibition of tumor angiogenesis by decreasing the ratio of circulating proangiogenic factors (e.g., VEGF) and antiangiogenic factors (e.g., thrombospondin-1, TSP-1), which are secreted in the tumor microenvironment in tumor-bearing mice and patients with cancer^3,7,10–14^. However, several clinical studies have revealed that these soluble factors do not reflect the therapeutic effects of LDMC^15,16^. Therefore, the antiangiogenic mechanism underlying LDMC efficacy remains largely unknown.

Regulated in development and DNA damage responses 1 (REDD1), initially identified as a transcriptional target of p53 following DNA damage and oxidative stress^17^, shows upregulated expression in response to various stress conditions elicited by hypoxia and chemotherapeutic drugs, including doxorubicin (DOX)^7,18–20^. A major function of REDD1 is the disruption of the inhibitory interaction between tuberous sclerosis complex-2 (TSC2) and 14-3-3 protein, subsequently inhibiting the mammalian target of rapamycin complex 1 (mTORC1) pathway^20^, which is a central signaling hub for controlling various cellular functions, including protein synthesis and angiogenesis^21,22^. Therefore, the available information suggests that REDD1 may negatively regulate tumor angiogenesis and lymphangiogenesis, although the underlying mechanism is not clear.

Here, we hypothesized that the antitumor effect of LDMC is associated with REDD1-dependent inhibition of tumor angiogenesis and lymphangiogenesis by targeting TECs. We found that REDD1 is essential for LDMC-mediated inhibition of tumor progression by attenuating tumor angiogenesis and lymphangiogenesis via reduced translation of *Vegfr-2/3*, providing new insights into the role of REDD1 in impairing TEC function.

## Materials and methods

### Cell culture and treatment

Primary human umbilical vein endothelial cells (HUVECs; ATCC® PCS-100-010) and human lymphatic endothelial cells (HLECs; #2500, ScienCell Research Laboratories, Inc., Carlsbad, CA) were maintained on fibronectin-coated cell culture plates in either endothelial cell medium (ScienCell Research Laboratories, Inc.) or Medium 199 (M119; HyClone, Logan, UT) supplemented with 20% fetal bovine serum (FBS). Mouse lung endothelial cells were prepared as previously described^23^. The cells were passaged at 80–90% confluence at a ratio of 1:2 and used for experiments at passages 3–5. Cells were treated for 24 h with DOX (3 nΜ), paclitaxel (PTX; 25 nM), or cisplatin (CisPt; 2 μM), which were purchased from Cayman Chemical Company (Ann Arbor, MI). Some cells were infected with *Redd1* (Ad-*Redd1*) or empty adenovirus (Ad-Control; Genenmed, Inc., Seoul, South Korea) at a multiplicity of infection (MOI) of 50 for 4 h or transfected with 80 nM small interfering RNA (siRNA), either the control (#SC-37007) or *Redd1* (#SC-45806; Santa Cruz Biotechnology, Dallas, TX), using Lipofectamine RNAiMAX (Invitrogen, Carlsbad, CA) according to the manufacturer’s instructions, followed by continuous culturing in fresh medium for 24 h. Tumor cells (HeLa, B16F1, and B16F10 cells) were cultured in Dulbecco’s modified Eagle’s medium supplemented with FBS (10%), penicillin (100 U/mL), and streptomycin (100 μg/mL).

### Cytotoxicity assay

Endothelial cells or tumor cells were seeded in 6-well plates coated with gelatin at a density of 2 × 10^5^ cells per well and maintained for 24 h, followed by treatment with different concentrations of anticancer drugs for 24 h. For evaluation of cytotoxicity, lactate dehydrogenase (LDH) release was assayed in cultured media using an LDH cytotoxicity detection kit (TaKaRa Bio, San Jose, CA) according to the manufacturer’s instructions. Cell viability was determined by a 3-(4,5-dimethyl-thiazol-2-yl)−2,5-diphenyltetrazolium bromide (MTT) colorimetric assay. MTT solution (100 µL of 5 mg/mL) in phosphate-buffered saline (PBS) was added to each well, which contained 1 mL of culture medium. After incubation for 4 h at 37 °C, 0.5 mL of dimethyl sulfoxide was added to dissolve the blue formazan products formed, and the absorbance was measured using a microplate reader at 550 nm. Cell viability was calculated as the percentage of the control absorbance.

### Assessment of in vitro angiogenesis and lymphangiogenesis

HUVECs and HLECs were seeded in 6-well plates coated with gelatin at a density of 1 × 10^5^ cells per well and maintained for 1 day. The cells were treated with or without 3 nΜ DOX for 24 h after transfection with or without the control or *Redd1* siRNA, followed by stimulation with 10 ng/mL VEGF-A (R&D Systems, Minneapolis, MN) or 10 ng/mL VEGF-C (R&D Systems) for the angiogenesis and lymphangiogenesis assays, respectively. The angiogenic properties of endothelial cells, such as migration and tube formation, were examined as described previously. Cell migration assays were performed using Boyden chambers. Tube-like structure formation was determined by image capture using a microscope. Quantification of endothelial cell migration and tube formation was determined using ImageJ software (NIH, Bethesda, MD).

### qRT-PCR analysis

HUVECs and HLECs were treated with or without 3 nΜ DOX for 24 h following transfection with or without the control or *Redd1* siRNA. Some cells were infected with Ad-control or Ad-Redd1 for 4 h and further incubated for 20 h. After treatment, total RNA was isolated from the cultured cells using TRIzol reagent (Invitrogen) and then used to synthesize first-strand cDNA using M-MLV Reverse Transcriptase (Promega, Madison, WI) according to the manufacturer’s instructions. qRT-PCR for determining *Vegfr-1/2/3*, *Igf-1r*, *Egfr*, *Redd1*, and *Gapdh* expression was performed using SYBR Real-Time qPCR 2X Master Mix (Elpis, Daejeon, South Korea) with the real-time PCR cycler Rotor-Gene Q (Qiagen, Germantown, MD). The following primers were used: 5′-GAACACAGCTCAAGCAAACC-3′ and 5′-GATCAAAGTGTCAAGTGGA-3′ for hs-*Vegf1*; 5′-TTTGGTTCTGTCTTCCAAAGT-3′ and 5′-ATGCTCAGCAGGATGGCAA-3′ for hs-*Vegfr-2*; 5′-AGATCTTGTCTGCTACAGCT-3′ and 5′-AGGGTCTTTGTAGATGTCCC-3′ for hs-*Vegfr-3*; 5′-GAAGGAGVAGATGACATTCC-3′ and 5′-GATCCTCAACTTGTGATCCG-3′ for hs-*Igf-1r*; 5′-CGTACCAGATGGATGTGAAC-3′ and 5′-CCGTCTTCCTCCATCTCATA-3′ for hs-*Egfr*; 5′-CGCCACAGTTTCCCGGAGGG-3′ and 5′-CCCTCCAAAATCAAGTGGGG-3′ for hs-*Redd1*; and 5′-CGCCACAGTTTCCCGGAGGG-3′ and 5′-CCCTCCAAAATCAAGTGGGG-3′ for hs-*Gapdh*.

### Western blotting

HUVECs and HLECs were suspended in RIPA buffer [50 mΜ Tris-HCl (pH 8.0), 150 mΜ NaCl, 1% Nonidet P-40, 0.5% deoxycholic acid, 0.1% sodium dodecyl sulfate (SDS)] and incubated on ice for 30 min for complete cell lysis. After centrifugation at 12,000 × *g* for 10 min, lysates (25 μg of protein) were separated by SDS-polyacrylamide gel electrophoresis and transferred to a nitrocellulose membrane, followed by Western blotting with antibodies against target proteins. Antibodies against mTOR (#2972), p-mTOR (#2971), S6K (#9202), p-S6K (#9205), 4E-BP1 (#9644), p-4E-BP1 (#2855), ERK (#9102), p-ERK (#9106), Akt (#9272), p-Akt (#9271), VEGFR-1 (#2893), VEGFR-2 (#2479), p-VEGFR-2 (#2478), EGFR (#4267), and IGF-1Rβ (#3027) were purchased from Cell Signaling Technology (Denver, CO). Other antibodies used included anti-β-actin (#A5441; Sigma-Aldrich, Burlington, MA), anti-REDD1 (#10638-1-AP; Proteintech, Rosement, IL), anti-VEGFR-3 (#AF349; R&D Systems), anti-p-VEGFR-3 (#CY111; Cell Applications, Inc., San Diego, CA), and goat anti-mouse IgG (#31430; Invitrogen), goat anti-rabbit IgG (#31460; Invitrogen), and rabbit anti-goat IgG (#31402; Invitrogen) secondary antibodies. Relative protein levels were quantified using ImageJ.

### Microarray analysis

Total and high-molecular-weight polysome-associated RNA was extracted using the RNeasy Micro Kit (#74004; Qiagen) from HUVECs infected with Ad-control or Ad-*Redd1*. The concentration, purity, and integrity of the extracted RNA were determined using NanoDrop 2000 spectrophotometry (Thermo Fisher Scientific, Waltham, MA). Microarray analysis of each RNA sample was performed by Macrogen (Seoul, South Korea) using the Affymetrix Human Gene 2.0 ST Array, and raw data preprocessing was also carried out by Macrogen. The fold change in angiogenesis-associated receptor gene transcripts in total and polysomal RNA samples was calculated by normalizing the signal intensities of the Ad-*Redd1*-infected cells to those of the Ad-control-infected cells.

### Polysome assay

HUVECs and HLECs (1 × 10^6^ cells) were transfected with 80 nM control or *Redd1* siRNA, followed by treatment with 3 nM DOX for 16 h. Some cells were infected with Ad-*Redd1* or Ad-control (MOI = 50) for 16 h. Cells were lysed in 400 μL of lysis buffer [15 mΜ Tris-HCl (pH 7.4), 0.3 Μ NaCl, 15 mΜ MgCl_2_, 0.1 mg/mL cycloheximide, 200 U Superase-In^TM^ (Ambion)]. After centrifugation at 2000 × *g* for 5 min, heparin (a broad-range RNase inhibitor) was added to the supernatant at a final concentration of 200 μg/mL. After removal of debris by centrifugation at 10,000 × *g* for 5 min, the cytosolic supernatants were overlaid on a 20–50% sucrose gradient (total volume: 5 mL) and centrifuged at 39,000 rpm for 120 min in a Beckman SW-55Ti rotor at 4 °C. After ultracentrifugation, gradients were fractionated in 0.2 mL aliquots with continuous monitoring of absorbance at 254 nm using the ISCO gradient fractionation system. High-molecular-weight polysomes were pooled, and RNA was extracted using TRIzol reagent (Invitrogen), followed by purification and concentration using an RNeasy PowerClean Pro Cleanup Kit (#13997–50; Qiagen). *Vegfr-1/2/3*, *Igf-1r*, and *Egfr* mRNA levels were analyzed using qRT-PCR with their specific primers.

### Assays with 5′-untranslated region (5′-UTR) luciferase reporters

The longest 5′-UTRs of human *Vegfr-1/2/3* and *Egfr* annotated in RefSeq (NCBI Reference Sequence Database; www.ncbi.nlm.nih.gov/RefSeq/) were cloned into the pGL4.13 vector (Promega) between the SV40 promoter and the *Firefly* luciferase open reading frame using HindIII (New England Biolabs, Ipswich, MA) and AvrII (New England Biolabs). Deletion mutants of a putative internal ribosome entry site (IRES) between -87 and -1 of the *Vegfr-1* 5′-UTR and an authentic IRES (core region: −56 to −20) between −56 and −1 of *Egfr* were also subcloned into the same cloning sites of the pGL4.13 vector. IRES sequences were identified via computational prediction using the IRESpy program (https://irespy.shinyapps.io/IRESpy/). HEK-293 cells were cotransfected with a pGL4.13/5′-UTR reporter construct and *Renilla* luciferase pGL4.74 vector as a normalization control reporter using Lipofectamine 3000 (Invitrogen), followed by treatment with or without DOX or infection with or without Ad-*Redd1*. Cell lysates were prepared 24 h later, and luciferase activity was measured using the Dual Luciferase Assay Kit (Promega). *Firefly* luciferase activity was normalized to *Renilla* activity.

### Experimental tumor model

Male C57BL/6 mice were obtained from Orient Bio, Inc. (Sungnam, South Korea), and *Redd1*^−/−^ mice were generated using CRISPR/Cas9 in C57BL/6 zygotes by ToolGen, Inc. (Seoul, South Korea). Mice were provided with a standard chow diet and tap water ad libitum and maintained under a 12:12-h dark-light cycle in a pathogen-free animal facility. Murine melanoma B16F1 (5 × 10^5^) and B16F10 (2 × 10^5^) cells suspended in 100 μL of saline were subcutaneously injected into the right dorsal flank of 6-week-old male WT and *Redd1*^−/−^ mice. After tumors were detected on Day 5, the mice were treated with saline or DOX (0.2 mg/kg/every other day, intraperitoneally). The tumor-bearing mice were treated with DOX for 10 or 27 days to analyze tumor tissues and mortality, respectively. Tumor growth was monitored every other day using a caliper, and mouse mortality was recorded every day. Tumor volume was calculated using the following formula: width^2^ × length × 0.52. Tumors were removed at the end of the experiment and fixed in 7.5% formaldehyde for paraffin embedding.

### Matrigel plug assay

Six-week-old male C57BL/6 and *Redd1*^−/−^ mice were injected with or without DOX (0.2 mg/kg/2 days, intraperitoneally). Matrigel (0.5 mL) containing 100 ng VEGF-A (or VEGF-C) and 10 U heparin was subcutaneously injected into mice 2 days after initial treatment with DOX under pentobarbital anesthesia (50 mg/kg, intraperitoneally). After 10 days, the mice were sacrificed by cervical dislocation, and the Matrigel plugs were carefully removed and photographed. Hemoglobin levels in the Matrigel plugs were measured using Drabkin’s Reagent Kit 525 (Sigma-Aldrich) to assess functional blood vessel formation. For identification of infiltrating vascular and lymphatic endothelial cells, immunohistochemistry was performed on slices of the Matrigel plug as described below.

### Analysis of immunohistochemical staining, vascular permeability, and hypoxic regions

Sections (10 μm) prepared from tumor tissues or Matrigel plugs were stained by incubating with primary antibodies for 2 h at room temperature, rinsing three times in PBS, and incubating with secondary antibody for 60–90 min at room temperature. The sections were mounted with Dako Fluorescent Mounting Medium (Dako North America, Inc., Glostrup, Denmark) after washing three times with PBS. Antibodies for immunostaining tumor tissues included anti-REDD1, anti-CD31 (#NB600–1475; Novus Biologicals, Centennial, CO), anti-VEGFR-2 (#SC-6251; Santa Cruz Biotechnology), anti-tyrosinase (#SC-20035; Santa Cruz Biotechnology), anti-VEGFR-3, anti-LYVE-1 (#11-034, AngioBio, San Diego, CA), anti-α-SMA (#M0851; Dako North America, Inc.), and anti-NG2 (#AB5320; Millipore, Burlington, MA) antibodies. Alexa Fluor 488-, Alexa Fluor 555-, or Alexa Texas Red-X-conjugated anti-rabbit, anti-rat, anti-mouse, and anti-goat secondary antibodies were obtained from Invitrogen. For assessment of hypoxic areas in tumors, hypoxyprobe^TM^-1 (solid pimonidazole hydrochloride, 60 mg/kg; #HP1–1000Kit; Hypoxyprobe, Burlington, MA) was intraperitoneally injected 60 min before perfusion fixation. The tumors were harvested, sectioned, and stained with FITC-labeled anti-hypoxyprobe-1 MAb1. Vascular permeability was determined using FITC-dextran (#FD500S; Sigma-Aldrich) as previously described^24^. Images were acquired and analyzed using a laser scanning confocal microscope with AiryScan (LSM 880; Zeiss, Jena, Germany). Colocalization between target proteins was assessed using Pearson’s correlation coefficient with ZEN software (version 2.3).

### TUNEL and caspase-3 activation assays

DNA fragmentation during apoptosis was detected using a TUNEL assay with the In Situ Cell Death Detection Kit (#12156792910; Roche, Indianapolis, IN). Paraffin-embedded sections were cleared with xylene and rehydrated according to the manufacturer’s instructions. After three washes in PBS, sections were preincubated inside a dark humidified chamber with 50 μL of TUNEL reaction mixture (5 μL of TUNEL-Enzyme solution + 45 μL of TUNEL-Label solution; tetramethylrhodamine-labeled dUTP) for 60 min at 37 °C and then counterstained with DAPI (#D9542; Sigma-Aldrich). The sections were mounted with mounting medium after washing three times with PBS. Immunofluorescence staining of active caspase-3 was performed with anti-caspase-3 antibody (#269518; R&D Systems) and Alexa Fluor 488-conjugated donkey anti-goat IgG (#A11055; Invitrogen), followed by counterstaining with DAPI. The sections were mounted with mounting medium after washing three times with PBS. Slides were imaged using the LSM 880. Quantification was performed using ZEN software.

### Statistical analysis

All experiments were performed independently at least four times. Statistical analysis was performed using GraphPad Prism (version 6.0; GraphPad Software, Inc., San Diego, CA). No statistical methods were used to predetermine the sample size. The experiments were randomized, investigators were blinded to allocation during experiments and outcome analysis, and no samples or animals were excluded from analysis. All values are presented as the mean ± standard deviation (SD). Statistical significance was determined by a two-tailed Mann–Whitney *U* test between two groups and one-way or two-way ANOVA with Holm-Sidak’s multiple comparisons test, depending on the experimental groups analyzed. Mouse survival was evaluated using the Kaplan–Meier method, and significant differences were analyzed using the log-rank test. Statistical significance was set at *P* < 0.05.

## Results

### Chemotherapeutic drugs induce REDD1 and downregulate VEGFR-2/3 expression

**Fig. 1:**
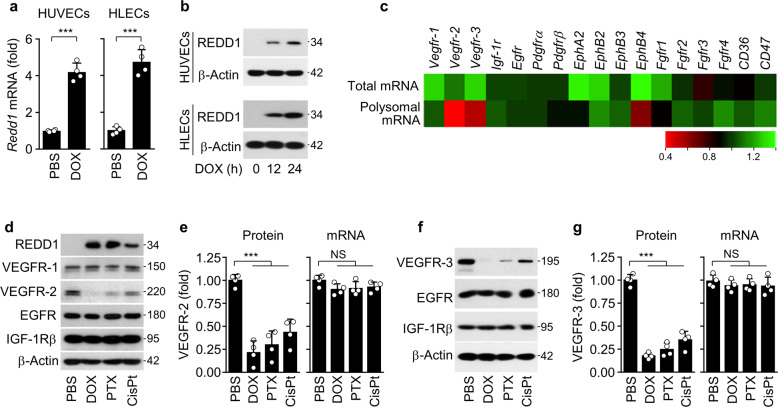
Chemotherapeutic drugs induce REDD1 and downregulate VEGFR-2/3 expression without altering their mRNA levels. **a**, **b** REDD1 mRNA and protein levels in the HUVECs and HLECs treated with doxorubicin (DOX; 3 nM) for 24 h. **c** Microarray analysis of total and polysomal mRNAs purified from the HUVECs infected with control adenovirus (Ad-C) or Ad-*Redd1* (Ad-R). Heat map of receptor genes involved in angiogenesis expressed as the fold change in total and polysomal mRNA levels in the Ad-*Redd1*-transfected HUVECs compared with the Ad-C-transfected cells (*n* = 3). **d** VEGFR-1, VEGFR-2, EGFR, and IGF-1Rβ expression levels in the HUVECs treated with DOX (3 nM), paclitaxel (PTX; 25 nM), or cisplatin (CisPt; 2 μM) for 24 h. **e** VEGFR-2 protein and mRNA levels in the HUVECs treated with chemotherapeutic drugs. **f**, **g** VEGFR-3, EGFR, and IGF-1Rβ expression levels (**f**) and VEGFR-3 expression at the protein and mRNA levels (**g**) in the HLECs treated with chemotherapeutic drugs for 24 h. Data are presented as the mean ± SD (*n* = 4). ***P < 0.001; NS, not significant.

To address the possible link between inhibition of tumor angiogenesis and increased REDD1 expression by LDMC^3,7^, we treated HUVECs and HLECs with noncytotoxic low-dose DOX (3 nM), whose concentration was determined by MTT-based mitochondrial activity and lactate dehydrogenase release assays (Supplementary Fig. 1a, b), and found a significant upregulation of REDD1 at the mRNA and protein levels (Fig. 1a, b). Since REDD1 can inhibit cap-dependent translation via mTORC1 inhibition^21^, we assessed the comparative profiles of total and polysomal mRNAs encoding angiogenesis-related receptors in HUVECs infected with Ad-control or Ad-*Redd1* using microarray analysis. REDD1 overexpression led to a prominent decrease in the *Vegfr-2/3* mRNA levels and a partial decrease in *EphB4* mRNA expression but did not affect the mRNA levels of other angiogenesis-related receptors, including *Vegfr-1*, in the high-molecular-weight polysomal fraction compared with total mRNA expression (Fig. 1c). In the HUVECs treated with a noncytotoxic dose of DOX (3 nM), PTX (25 nM), or CisPt (2 μM), Western blot analysis confirmed a decrease in the expression of VEGFR-2 but not VEGFR-1, epidermal growth factor receptor (EGFR), or insulin-like growth factor-1 receptor β (IGF-1Rβ) without altering mRNA expression (Fig. 1d, e; Supplementary Fig. 1a, b). Similarly, we found that the expression of VEGFR-3, but not EGFR and IGF-1Rβ, decreased without altering the mRNA levels in the HLECs treated with the same chemotherapeutic drugs (Fig. 1f, g). These results suggest that noncytotoxic doses of DOX, PTX, and CisPt inhibit VEGFR-2/3 expression via post-transcriptional regulation.

### Low-dose DOX impairs *Vegfr-2/3* translation and endothelial dysfunction via REDD1 induction

**Fig. 2:**
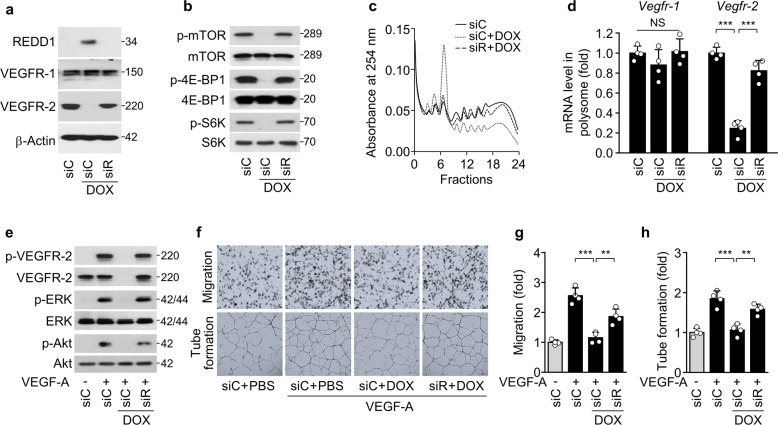
Low-dose DOX impairs *Vegfr-2* translation and angiogenesis via REDD1 induction. VEGFR-1/2 expression and VEGF-A-induced angiogenesis were examined in HUVECs treated with DOX following transfection with control (siC) or *Redd1* siRNA (siR). **a**, **b** Western blots of REDD1 and VEGFR-1/2 (**a**) as well as phosphorylated mTOR, 4E-BP1, and S6K (**b**). **c** Polysome profiling using sucrose density gradient ultracentrifugation. **d** qRT-PCR of *Vegfr-1/2* mRNAs associated with high-molecular-weight polysomes (*n* = 4). **e** Western blots of phosphorylated VEGFR-2, ERK, and Akt in the HUVECs stimulated with VEGF-A. **f** Representative images of the migration (upper) and tube formation (lower) of the HUVECs stimulated with VEGF-A were obtained using Boyden chamber and Matrigel-based morphogenesis assays, respectively. **g**, **h** Quantitation of migration (**g**) and tube formation (**h**) was performed using ImageJ software (*n* = 4). Data are presented as the mean ± SD. **P < 0.01, ***P < 0.001; NS, not significant.

Given that REDD1 inhibits the mTORC1-mediated translational pathway^20,21^, we explored the possible involvement of REDD1 in the suppression of VEGFR expression in endothelial cells exposed to DOX as a representative chemotherapeutic drug. siRNA-mediated *Redd1* knockdown rescued the DOX-induced repression of VEGFR-2/3 expression but did not alter VEGFR-1 and IGF-1Rβ expression in HUVECs and HLECs (Fig. 2a; Supplementary Fig. 2a). Moreover, *Redd1* knockdown blocked DOX-mediated inhibition of mTORC1-dependent phosphorylation of 4E-BP1 and S6K (Fig. 2b; Supplementary Fig. 2b), which are regulators that coordinate translational initiation and protein synthesis at the 5′-UTR of mRNA^22^. Consistent with this finding, *Redd1* knockdown rescued the DOX-induced decrease in high-molecular-weight polysome formation in both types of endothelial cells (Fig. 2c; Supplementary Fig. 2c) and the polysome-associated mRNA levels of *Vegfr-2/3*; however, the Vegfr-1 and *Egfr* mRNA levels were not altered under any conditions (Fig. 2d; Supplementary Fig. 2d). This finding suggests that DOX-induced REDD1 causes translational repression of *Vegfr-2/3* mRNAs by inhibiting the mTORC1 pathway. We next examined whether DOX impairs the angiogenic and lymphangiogenic abilities of endothelial cells through REDD1-dependent downregulation of VEGFR-2/3 expression. DOX treatment resulted in a significant inhibition of the angiogenic and lymphangiogenic signaling pathways (ERK and Akt phosphorylation) and behaviors (migration and tube formation) of HUVECs and HLECs in response to VEGF-A and VEGF-C, respectively (Fig. 2e–h; Supplementary Fig. 2e, f). These results suggest that low-dose DOX selectively decreases *Vegfr-2/3* translation and impairs the angiogenic and lymphangiogenic function of vascular and lymphatic endothelial cells via REDD1-induced mTORC1 pathway inhibition. Because REDD1 has a short half-life of 5–10 min^25^, we further examined the time-course expression profiles of REDD1 and VRGFR-2 in HUVECs following single or repeated treatment with low-dose DOX, which is considered an in vitro model of conventional chemotherapy or LDMC (Supplementary Fig. 3a). Single DOX treatment resulted in REDD1 induction and downregulation of VEGFR-2 expression at 24 h, which thereafter recovered to control levels; however, repeated treatment with DOX every 24 h maintained REDD1 expression along with VEGFR-2 suppression until 72 h (Supplementary Fig. 3b). This finding suggests that maintenance of REDD1 expression during repeated DOX treatment is a therapeutic advantage of a metronomic regimen.

### REDD1 overexpression leads to translational repression of *Vegfr-2/3* mRNAs

**Fig. 3:**
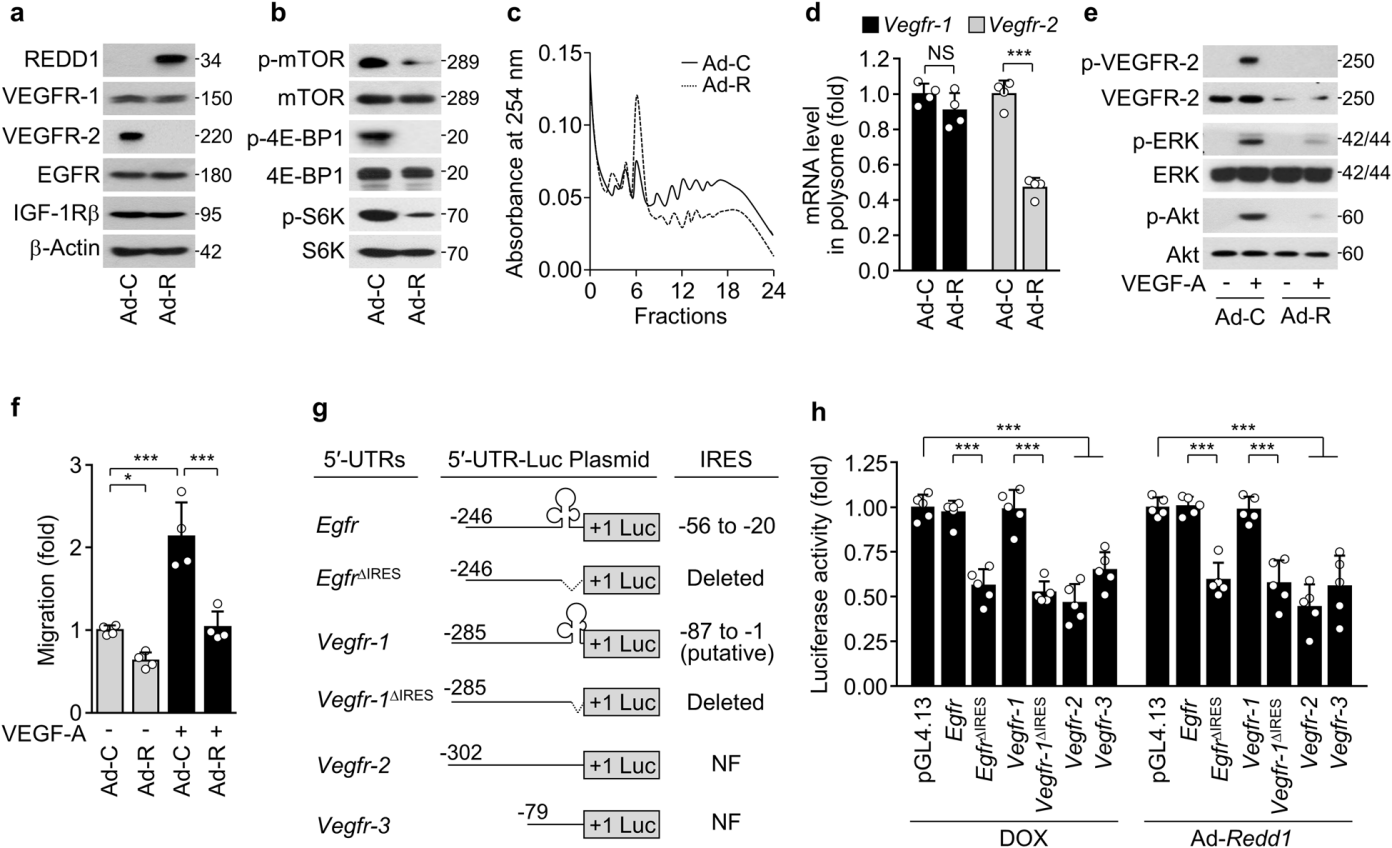
REDD1 overexpression suppresses *Vegfr-2* translation and angiogenesis. **a**–**f** VEGFR-1/2 expression and VEGF-A-induced angiogenesis were examined in the HUVECs infected with control adenovirus (Ad-C) or Ad-*Redd1* (Ad-R) at an MOI of 50. **a**, **b** Western blots of VEGFR-1/2, EGFR, and IGF-1Rβ (**a**) as well as phosphorylated mTOR, 4E-BP1, and S6K (**b**). **c** Polysome profiling using sucrose density gradient ultracentrifugation. **d** qRT-PCR of *Vegfr-1/2* mRNAs associated with high-molecular-weight polysomes (*n* = 4). **e** Western blots of phosphorylated VEGFR-2, ERK, and Akt in the HUVECs stimulated with VEGF-A. **f** Quantitative migration and tube formation of HUVECs in response to VEGF-A were assessed using Boyden chamber and Matrigel-based morphogenesis assays, respectively. (*n* = 4). **g** Schematic representation of the 5′-UTRs of *Egfr*, *Vegfr-1*, *Vegfr-2*, and *Vegfr-3*; clover-like structures in the 5′-UTRs of *Egfr* (-54 to -25) and *Vegfr-1* (−87 to −1) indicate authentic IRES and putative IRES sequences, respectively; +1 indicates the AUG start codon, and dotted lines indicate deleted regions of 5′-UTRs. NF, not found. **h** HEK-293 cells were cotransfected with the pGL4.13/5′-UTR-*Firefly* luciferase construct and *Renilla* luciferase pGL4.74 vector, followed by treatment with or without DOX or infection with Ad-C or Ad-*Redd1*. Reporter activities were measured in cell lysates using a Dual Luciferase Assay Kit (*n* = 5). Data are presented as the mean ± SD. *P < 0.05, ***P < 0.001; NS, not significant.

We next examined whether REDD1 dysregulates endothelial cell function through mTORC1-mediated translational repression of *Vegfr-2/3* mRNAs. Compared with control adenovirus expression, adenoviral overexpression of REDD1 decreased VEGFR-2 expression without affecting VEGFR-1, EGFR, and IGF-1Rβ expression in HUVECs (Fig. 3a) and downregulated the expression of VEGFR-3, but not EGFR and IGF-1Rβ, in HLECs (Supplementary Fig. 4). As expected, REDD1 overexpression inhibited mTOR, 4E-BP1, and S6K phosphorylation (Fig. 3b); the assembly of high-molecular-weight polysome complexes (Fig. 3c); and the polysome-associated mRNA levels of *Vegfr-2*, but not *Vegfr-1*, in HUVECs compared with control cells (Fig. 3d). Consequently, REDD1 overexpression suppressed VEGF-A-induced angiogenic signaling and migration of HUVECs (Fig. 3e, f). These results suggest that REDD1 causes translational repression of *Vegfr-2/3* mRNAs in vascular and lymphatic endothelial cells by inhibiting the mTORC1 pathway.

mTORC1 inhibition has been shown to attenuate translation of mRNA transcripts containing the common cap structure of 7-methyl-GTP at the end of their 5′-UTRs or 5′-terminal oligopyrimidine (TOP) motifs located immediately after the mRNA cap structure but not of mRNAs with an IRES within the 5′-UTR^25,26^. Thus, we analyzed whether there were IRES and TOP sequences in the 5′-UTRs of all three *Vegfr* mRNAs. Computational analysis using IRESpy identified a putative IRES sequence spanning nucleotides −87 to −1 upstream of the initiator ATG in the 5′-UTR of *Vegfr-1* mRNA but not in *Vegfr-2/3* transcripts (Fig. 3g); moreover, 5′-TOP motifs were not identified in any of the three *Vegfr* transcripts. Thus, we performed 5′-UTR luciferase reporter assays of wild-type (WT) and IRES-deleted 5′-UTRs of *Vegfr-1/2/3* and *Egfr* as a positive control of the IRES-containing 5′-UTR^27^. DOX treatment or REDD1 overexpression inhibited the 5′-UTR reporter activity of the putative IRES-deleted *Vegfr-1*, *Vegfr-2*, *Vegfr-3*, and IRES-deleted *Egfr* sequences but not of the WT *Vegfr-1* or *Egfr* sequences (Fig. 3h). These results suggest that REDD1 induced by DOX selectively represses the transcription of *Vegfr-2/3* but not *Vegfr-1* or *Egfr* via mTORC1 inhibition.

### REDD1 is essential for metronomic DOX-induced antiangiogenic and antilymphangiogenic effects

**Fig. 4:**
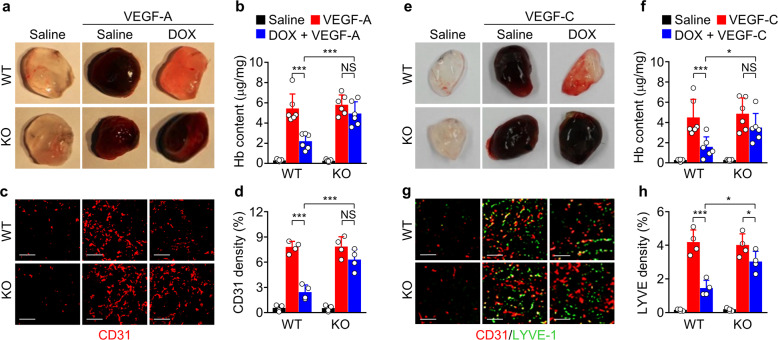
Metronomic DOX treatment inhibits in vivo angiogenesis and lymphangiogenesis in the WT but not *Redd1*^−/−^ mice. **a** Representative images of Matrigel plugs containing saline or VEGF-A removed from the WT and *Redd1*^**−/−**^ mice metronomically treated with or without DOX. **b** Quantitated levels of hemoglobin (Hb) extracted from the plugs (*n* = 6). **c** Representative immunostaining of CD31^+^ vessels in a Matrigel plug section. Scale bar, 100 μm. **d** Quantitated levels of CD31^+^ vessels in the plugs (*n* = 4). **e** Representative images of Matrigel plugs containing saline or VEGF-C removed from the WT and *Redd1*^**−/−**^ mice metronomically treated with or without DOX. **f** Quantitated levels of Hb extracted from the plugs (*n* = 6). **g** Representative immunostaining of CD31^+^ and LYVE-1^+^ vessels in a Matrigel plug section. Scale bar, 100 μm. **h** Quantitated levels of LYVE-1^+^ lymphatic vessels (*n* = 4). Data are presented as the mean ± SD. *P < 0.05, ***P < 0.001; NS, not significant.

We further explored the link between REDD1 and metronomic DOX treatment in the context of angiogenesis and lymphangiogenesis using WT and *Redd1-*deficient (*Redd1*^−/−^) mice, which were generated using the CRISPR/Cas9 system (Supplementary Fig. 5a–c). We confirmed that low-dose DOX treatment resulted in upregulation of REDD1 expression, inhibition of mTORC1-dependent 4E-BP1 and S6K phosphorylation, and downregulation of VEGFR-2 expression without altering VEGFR-1, EGFR, and IGF-1Rβ expression in lung endothelial cells isolated from the WT mice but not those from the *Redd1*^−/−^ mice compared with the untreated controls (Supplementary Fig. 5d–f). Next, we employed a Matrigel plug assay to further investigate the role of REDD1 in in vivo angiogenesis and lymphangiogenesis. Matrigel plugs containing VEGF-A, which were implanted subcutaneously into the saline-treated control WT and *Redd1*^−/−^ mice, exhibited a dark-red color indicative of neovascularization, increased hemoglobin content, and enhanced CD31^+^ microvessel density. These features were not observed in Matrigel plugs from the WT mice but were maintained in the *Redd1*^−/−^ mice after metronomic DOX treatment (Fig. 4a–d). Similarly, Matrigel plugs containing VEGF-C showed both increased angiogenesis (dark red color) and increased LYVE-1^+^ lymphatic vessel density in the control WT and *Redd1*^−/−^ mice, but these changes were again suppressed in the WT mice but not the *Redd1*^−/−^ mice after metronomic DOX treatment (Fig. 4e–h). These results indicate that metronomic DOX treatment inhibits in vivo angiogenic and lymphangiogenic functions in vascular and lymphatic endothelial cells in a REDD1-dependent manner.

### *Redd1* loss impairs LDMC-mediated inhibition of tumor growth, angiogenesis, and lymphangiogenesis

**Fig. 5:**
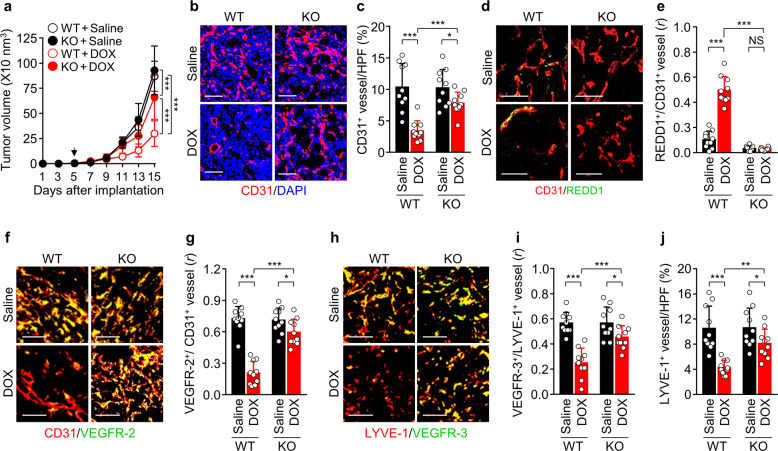
LDMC with DOX inhibits tumor growth, angiogenesis, and lymphangiogenesis in B16F1 tumor-bearing WT but not *Redd1*^−/−^ mice. **a** Comparison of tumor growth in the WT and *Redd1*^−/−^ mice metronomically treated with saline or DOX (*n* = 10 per group). The arrow indicates the start of DPX treatment. **b** Representative images of tumor sections showing CD31^+^ blood vessels and DAPI-stained nuclei. Scale bar, 100 μm. **c** Quantification of CD31^+^ blood vessel density per high-power field (HPF) (*n* = 10). **d** Representative images of tumor sections showing CD31^+^ blood vessels and REDD1 expression. Scale bar, 50 μm. **e** Quantification of their colocalization (*n* = 10). **f** Representative images of tumor sections showing CD31^+^ vessels and VEGFR-2 expression. Scale bar, 100 μm. **g** Quantification of their colocalization (*n* = 10). **h** Representative images of tumor sections showing LYVE-1^+^ lymphatic vessels and VEGFR-3 expression. Scale bar, 100 μm. **i** Quantification of colocalization of VEGFR-3 and LYVE-1 (*n* = 10). **j** Quantification of LYVE-1^+^ lymphatic vessel density (*n* = 10). Data are presented as the mean ± SD. *P < 0.05, **P < 0.01, ***P < 0.001; NS, not significant. *r*, Pearson’s correlation coefficient.

We next investigated whether REDD1 is a determinant of LDMC-mediated endothelial dysfunction and tumor growth inhibition in a B16F1 melanoma tumor-bearing mouse model (Supplementary Fig. 6a). Although there were no differences in tumor growth between the untreated WT and *Redd1*^−/−^ mice, LDMC with DOX significantly inhibited tumor growth in both the WT mice and the *Redd1*^−/−^ mice and was more effective in the WT mice (65.6%) than in the *Redd1*^−/−^ mice (23.9%) compared with the untreated mice (Fig. 5a; Supplementary Fig. 6b). The reduced efficacy in the *Redd1*^−/−^ mice was correlated with significant decreases in CD31^+^ tumor vessel density, caspase-3 activation, and TUNEL-positive apoptosis (Fig. 5b and c; Supplementary Fig. 6c–f). LDMC with DOX increased REDD1 expression in CD31^+^ tumor vessels only in the WT mice (Fig. 5d, e), consequently inhibiting tumor endothelial VEGFR-2 expression in the WT but not *Redd1*^−/−^ mice (Fig. 5f, g); however, DOX treatment did not significantly alter serum VEGF levels in the tumor-bearing WT and *Redd1*^−/−^ mice (Supplementary Fig. 7). Metronomic DOX treatment also resulted in a significant decrease in lymphatic endothelial VEGFR-3 expression and LYVE-1^+^ tumor lymphatic vessel density in the WT but not *Redd1*^−/−^ mice (Fig. 5h–j). Collectively, the results suggest that LDMC with DOX effectively inhibits tumor growth via REDD1-dependent suppression of tumor endothelial VEGFR-2/3 expression, angiogenesis, and lymphangiogenesis.

### *Redd1* loss prevents LDMC-induced tumor vessel normalization

**Fig. 6:**
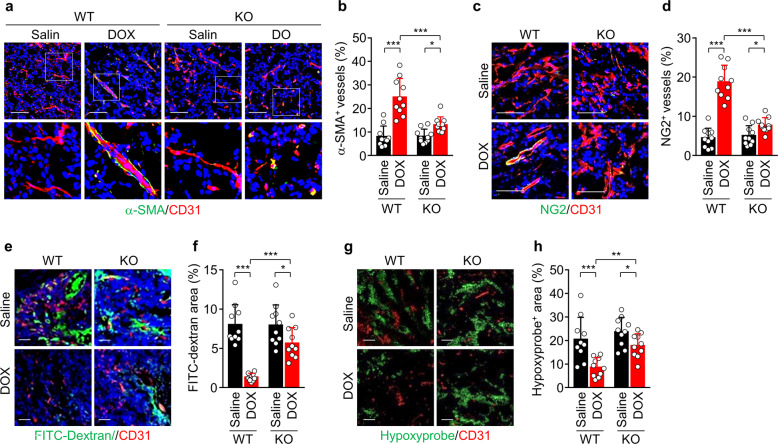
LDMC with DOX improves tumor vessel normalization in the WT but not *Redd1*^−/−^ mice. **a** Representative images of α-SMA^+^ smooth muscle cell coverage on CD31^+^ tumor vessels in B16F1 tumors. Scale bar, 50 μm. **b** Quantification of α-SMA^+^ smooth muscle cell coverage (*n* = 10). **c** Representative images of NG2^+^ pericyte coverage on CD31^+^ tumor vessels. Scale bar, 50 μm. **d** Quantification of NG2^+^ pericyte coverage (*n* = 10). **e** Representative images of tumor sections showing FITC-dextran leakage, CD31^+^ blood vessels, and DAPI-stained nuclei. Scale bar, 50 μm. **f** Quantification of FITC-dextran leakage (*n* = 10). **g** Representative images of tumor sections showing the hypoxyprobe^+^ area and CD31^+^ blood vessels. Scale bar, 50 μm. **h** Quantification of hypoxyprobe^+^ area (*n* = 10). Data are presented as the mean ± SD. *P < 0.05, **P < 0.01, ***P < 0.001.

The VEGF/VEGFR-2 system induces an abnormal tumor vessel structure with enhanced vascular permeability, resulting in decreased drug delivery and efficacy^28^. Thus, we examined whether LDMC-induced REDD1 improves tumor vascular permeability and normalization. LDMC with DOX increased the recruitment of α-SMA^+^ smooth muscle cells and NG2^+^ pericytes, which are essential for maintaining vascular integrity and normalization^29^, to tumor blood vessels in the WT but not *Redd1*^−/−^ mice (Fig. 6a–d). As a result, DOX treatment effectively reduced FITC-dextran leakage and hypoxic areas in tumors of the WT mice compared with those of the *Redd1*^−/−^ mice (Fig. 6e–h). Altogether, these results suggest that metronomic DOX treatment improves tumor vessel normalization and subsequently its therapeutic efficacy via upregulation of REDD1 expression.

### *Redd1* loss impairs LDMC-mediated suppression of mouse mortality and tumor metastasis

**Fig. 7:**
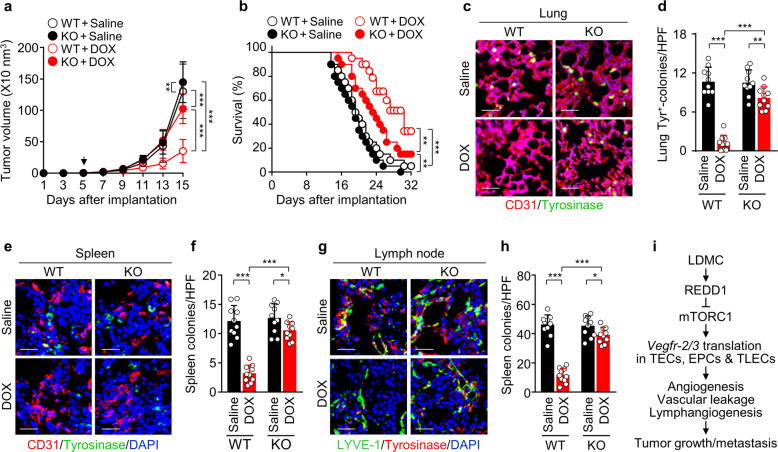
LDMC with DOX inhibits tumor progression and metastasis in B16F10 tumor-bearing WT but not *Redd1*^−/−^ mice. **a** Comparison of tumor growth in the B16F10 tumor-bearing WT and *Redd1*^−/−^ mice metronomically treated with saline or DOX (*n* = 12 per group). **b** Kaplan–Meier survival curves of the WT and *Redd1*^−/−^ mice treated with saline or metronomic DOX (*n* = 20 per group). **c** Representative images of lung-metastatic tyrosinase^+^ colonies and CD31^+^ blood vessels. Scale bar, 50 μm. **d** Quantification of tyrosinase^+^ colonies per high-power field (HPF) (*n* = 10). **e** Representative images of spleen-metastatic tyrosinase^+^ colonies and CD31^+^ blood vessels. Scale bar, 50 μm. **f** Quantification of tyrosinase^+^ colonies (*n* = 10). **g** Representative images of lymph node-metastatic tyrosinase^+^ colonies and LYVE-1^+^ lymphatic vessels. Scale bar, 50 μm. **h** Quantification of tyrosinase^+^ colonies (*n* = 10). **i** Diagram depicting inhibition of tumor angiogenesis, lymphangiogenesis, vessel permeability, growth, and metastasis by LDMC-induced REDD1 expression and subsequent translational repression of *Vegfr-2/3* mRNAs in tumor vascular and lymphatic endothelial cells (TVECs and TLECs). Data, except for those for the mouse survival curve (**b**), are presented as the mean ± SD. *P < 0.05, **P < 0.01, ***P < 0.001.

As tumor angiogenesis and lymphangiogenesis are correlated with not only tumor growth but also metastasis, we examined the role of REDD1 in the therapeutic effects of LDMC in a mouse metastatic model of B16F10 melanoma. LDMC with DOX resulted in a significant inhibition of B16F10 tumor growth in the WT mice compared with the *Redd1*^−/−^ mice (Fig. 7a), similar to the B16F1 tumor model results. Consistent with this result, DOX treatment increased the overall survival rate of the tumor-bearing WT and *Redd1*^−/−^ mice compared with the untreated controls, and its efficacy was much higher in the WT mice than in the *Redd1*^−/−^ mice (Fig. 7b). Moreover, DOX treatment resulted in more effective inhibition of metastatic colony formation in the lungs, spleen, and lymph nodes in the WT mice than in the *Redd1*^−/−^ mice (Fig. 7c–h). These data indicate that LDMC with DOX suppresses tumor metastasis and improves mouse survival in a REDD1-dependent manner.

## Discussion

LDMC has been shown to inhibit tumor angiogenesis and consequently tumor progression by selectively and specifically targeting activated TECs among normal and tumor cells without severe side effects^3,7^. However, the molecular mechanism underlying the therapeutic effects of LDMC in the context of endothelial cell function has not been fully elucidated. We found that LDMC dysregulates endothelial cell function by downregulating VEGFR-2/3 expression, subsequently attenuating tumor growth and metastasis by suppressing angiogenesis and lymphangiogenesis and improving tumor vessel normalization. Reduced VEGFR-2/3 expression was not correlated with their mRNA levels but was rather highly correlated with a decrease in translational efficiency via LDMC-mediated induction of REDD1 expression and inhibition of the mTORC1 pathway. Thus, LDMC-mediated TEC dysfunction is directly linked to the translational repression of *Vegfr-2/3* mRNA transcripts.

Accumulating evidence suggests that LDMC exerts both direct and indirect effects on endothelial cell function. The direct effect may be associated with the intrinsically high sensitivity of proliferating TECs to metronomic chemotherapy;^5,8,10^ moreover, LDMC does not impair normal endothelial and tumor cell functions^3,7^. The indirect effect may result from differential expression of pro- and antiangiogenic factors, such as decreased VEGF-A or elevated TSP-1 and soluble VEGFR-1 expression, in tumor-bearing mice and cancer patients administered LDMC^7,10–12^. However, a randomized phase II trial showed no significant difference in plasma TSP-1 levels between responders and nonresponders among patients receiving LDMC with cyclophosphamide^15,16^, suggesting that TSP-1 is not consistently involved in the therapeutic response of LDMC. Moreover, we found that LDMC with DOX did not alter VEGF plasma levels in tumor-bearing mice, probably due to the presence of an IRES in the 5′-UTR of the VEGF transcript^30^. Consistent with our results, several studies showed that LDMC with trofosfamide or CisPt and docetaxel did not change plasma VEGF levels in patients with various cancers^15,16^. Notably, metronomic administration of chemotherapeutic agents (cyclophosphamide, PTX, and CisPt) suppresses exogenously administered VEGF-A-mediated angiogenesis in tumor-free rats^31^. This finding suggests that LDMC may downregulate the expression of VEGFRs in endothelial cells rather than inhibit VEGF production. In line with this notion, a recent study on a breast tumor-bearing mouse model showed that metronomic PTX treatment suppresses VEGFR-2 expression, tumor angiogenesis, and lymphatic vessel formation, although the authors did not examine the expression levels of VEGFR-3, a lymphangiogenic receptor^8^. Taking these results and ours into consideration, we can postulate that downregulation of VEGFR-2/3 expression is crucial for the antiangiogenic therapeutic effect of LDMC.

REDD1 acts as an endogenous mTORC1 inhibitor^32^, resulting in inhibition of mTORC1-mediated mRNA translation^20,33^. Our data demonstrated that low-dose DOX treatment or REDD1 overexpression resulted in translational repression of *Vegfr-2/3* mRNAs and subsequently suppressed angiogenesis and lymphangiogenesis by preventing mRNA-bound polysome complex assembly. However, the effects of DOX were attenuated in *Redd1* knockdown cells. These results suggest that DOX-induced REDD1 expression in TECs suppresses VEGFR-2/3 expression by inhibiting the mTORC1-mediated, cap-dependent, translational mechanism. Notably, the mTORC1 inhibitor rapamycin was shown to reduce tumor growth by suppressing the angiogenic response of endothelial cells to exogenous VEGF-A, suggesting that the mTORC1 pathway downregulates the expression of VEGFRs^34^. Indeed, several studies have demonstrated that the therapeutic effects of rapamycin on angiogenesis, lymphangiogenesis, and tumor growth are associated with the downregulation of VEGFR-2/3 expression in endothelial cells^35–37^. These results are consistent with our findings and support our hypothesis that LDMC induces endothelial cell dysfunction through translational repression of *Vegfr-2/3* mRNA by upregulating REDD1 expression.

The major question that arises from our work is how REDD1 selectively decreases proangiogenic receptor gene expression. Considering its function as an mTORC1 inhibitor, REDD1 shows translational repression of a subset of mRNAs but not the overall translational program; this process is similar to that of rapamycin, where some mRNAs either with a 5′-TOP sequence or without an IRES are silenced in a cap-dependent manner through inhibition of the mTORC1 pathway^26^. REDD1, induced by LDMC, can control cell fate by reprogramming the translational initiation machinery from a cap-dependent to an IRES-dependent system. Indeed, we found that metronomic DOX treatment suppressed mTORC1-dependent translation of angiogenic *Vegfr-2/3* mRNAs but not *Igf-1r* and *Egfr* mRNAs containing an IRES^27,38^, thereby attenuating tumor growth and metastasis. We also found that REDD1 does not repress *Vegfr-1* mRNA translation due to the existence of an IRES in its 5′-UTR. These findings suggest that LDMC switches the angiogenic and lymphangiogenic state of TECs to angiostatic and lymphangiostatic phenotypes via REDD1-mediated VEGFR-2/3 biosynthesis.

The VEGF/VEGFR-2 axis is a key player in the formation of tumor blood vessels with abnormal structure and function, increasing spatially uneven vessel permeability and interstitial fluid pressure as well as hindering drug delivery^28^. These characteristics promote tumor progression and resistance to chemotherapy. Therefore, pharmacological inhibitors of the VEGF/VEGFR-2 pathway inhibit tumor angiogenesis to limit tumor growth by blocking the nutrient and oxygen supply and restore structural and functional vascular integrity, termed vascular normalization, leading to improved drug delivery and therapeutic efficacy. Indeed, we found that LDMC with DOX inhibited VEGFR-2 expression, tumor angiogenesis, and vascular abnormalities, resulting in suppression of vascular leakage, tumor hypoxic areas, tumor growth, and metastasis in the WT mice compared with the *Redd1*^−/−^ mice. Consistent with our findings, recent studies suggested that metronomic regimens can normalize tumor vasculature, as predicted by mathematical models and as evidenced by improved tumor perfusion and reduced tumor hypoxia in a xenograft mouse model of pancreatic cancer^39,40^. These results indicate that REDD1 functions as a novel regulator of tumor vessel remodeling and normalization in the tumor microenvironment by downregulating VEGFR-2 expression in TECs during LDMC.

The VEGF-C/VEGFR-3 pathway stimulates tumor lymphangiogenesis, which acts as a conduit for tumor dissemination and distant metastasis^41^. Thus, an antilymphangiogenic regimen targeting VEGFR-3 is considered an effective strategy for attenuating tumor metastasis^42^. However, there is little research on the effects of LDMC on intratumoral lymphangiogenesis. Nevertheless, indirect evidence indicates that LDMC inhibits tumor lymphangiogenesis associated with distant metastasis. LDMC with PTX has been shown to suppress intratumoral lymphangiogenesis in breast tumor-bearing mice, although its underlying mechanism was not elucidated^8^. In the present study, we demonstrated that LDMC with DOX suppressed translational biosynthesis of VEGFR-3 and tumor lymphangiogenesis, significantly reducing lymphatic metastasis in the WT mice but not the *Redd1*^−/−^ mice. These results suggest that LDMC-induced REDD1 is a negative regulator of tumor lymphangiogenesis and lymphatic metastasis in tumor-bearing mice, eventually improving overall mouse survival.

Proliferative endothelial cells supporting tumor vessels, but not tumor cells and other normal cells, are considered the primary target of LDMC, which can, in part, suppress tumor cell proliferation. TECs are different from normal endothelial cells in terms of pathophysiology and therapy^43^. TECs show dynamic features, including highly proliferative behavior in the tumor microenvironment, while normal endothelial cells remain in a quiescent or static state, allowing regulation of intrinsic vascular function. TECs from highly metastatic tumors, compared with those from other metastatic tumors and normal endothelial cells, exhibit more angiogenic properties in response to proangiogenic factors, including VEGF. Although TECs originate from endothelial cells present in the normal vasculature, there are molecular differences between them, such as differential gene expression profiles, including increased VEGFR-2 levels in TECs^43^. For these reasons, TECs can easily lose their intrinsic angiogenic activity when exposed to low doses of anticancer drugs. Although the molecular targets involved in the high sensitivity of TECs to LDMC remain unknown, our results provide evidence that REDD1 plays a crucial role in impairing the angiogenic functions of TECs exposed to LDMC.

In conclusion, our study demonstrates that LDMC-induced REDD1 causes functional dysregulation of tumor-associated vascular and lymphatic endothelial cells through selective translational repression of *Vegfr-2/3* mRNAs, which in turn inhibits the formation of tumor blood and lymphatic vascular networks and promotes tumor vascular normalization and a favorable tumor microenvironment (Fig. 7i). Thus, the REDD1-mTORC1-VEGFR-2/3 axis is crucial for LDMC-induced tumor therapeutic efficacy and supports the notion that REDD1 is an LDMC-sensitive molecular sensor that modulates phenotypic switching of endothelial cells from angiogenic and lymphangiogenic phenotypes to angiostatic and lymphangiostatic states. These findings also suggest that REDD1 is a new therapeutic target for the treatment of solid tumors.

## Supplementary information

Highlights

Graphic abstract

Supplementary Information
