# Supplementary material for: REDD1 is a determinant of low-dose metronomic doxorubicin-elicited endothelial cell dysfunction through downregulation of VEGFR-2/3 expression
Source: Exp Mol Med. 2021 Oct 25;53(10):1612–22. doi: 10.1038/s12276-021-00690-z (PMC8568908; doi:10.1038/s12276-021-00690-z)
Supplement: Supplementary file 1 — Highlights [file 12276_2021_690_MOESM1_ESM.docx]

**Highlights**

- LDMC induces REDD1 expression in tumor-associated endothelial cells.
- REDD1 represses VEGFR-2/3 biosynthesis, tumor angiogenesis, and lymphangiogenesis.
- Loss of REDD1 prevents LDMC-induced tumor growth and metastasis.
- REDD1 is an LDMC molecular sensor and is a therapeutic target for solid tumors.
