# Supplementary figures and images for: REDD1 is a determinant of low-dose metronomic doxorubicin-elicited endothelial cell dysfunction through downregulation of VEGFR-2/3 expression
Source: Exp Mol Med. 2021 Oct 25;53(10):1612–22. doi: 10.1038/s12276-021-00690-z (PMC8568908; doi:10.1038/s12276-021-00690-z)

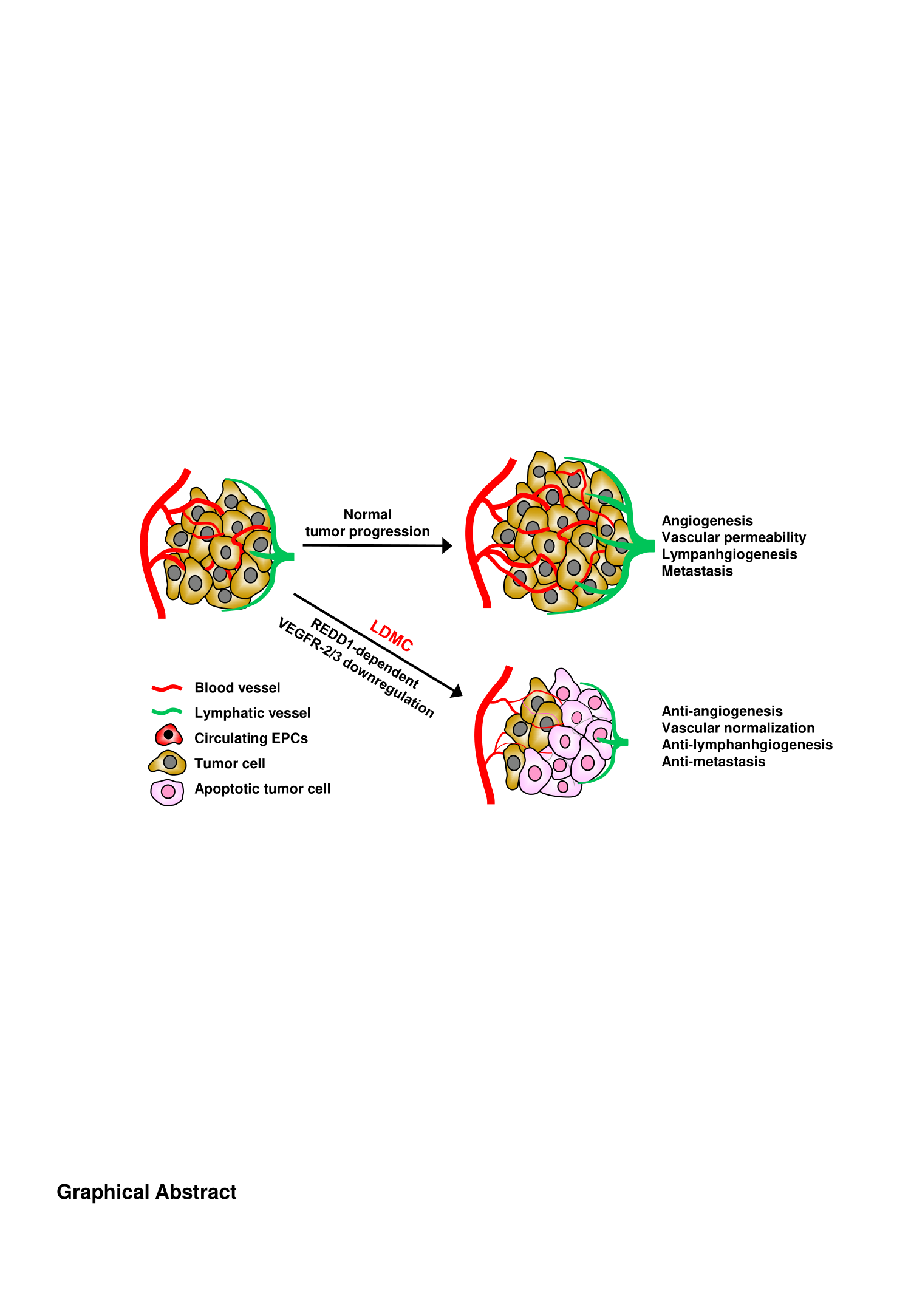

Supplement: Supplementary file 2 — Graphic abstract [file 12276_2021_690_MOESM2_ESM.tif]
