# Supplementary material for: REDD1 is a determinant of low-dose metronomic doxorubicin-elicited endothelial cell dysfunction through downregulation of VEGFR-2/3 expression
Source: Exp Mol Med. 2021 Oct 25;53(10):1612–22. doi: 10.1038/s12276-021-00690-z (PMC8568908; doi:10.1038/s12276-021-00690-z)
Supplement: Supplementary file 3 — Supplementary Information [file 12276_2021_690_MOESM3_ESM.pdf]

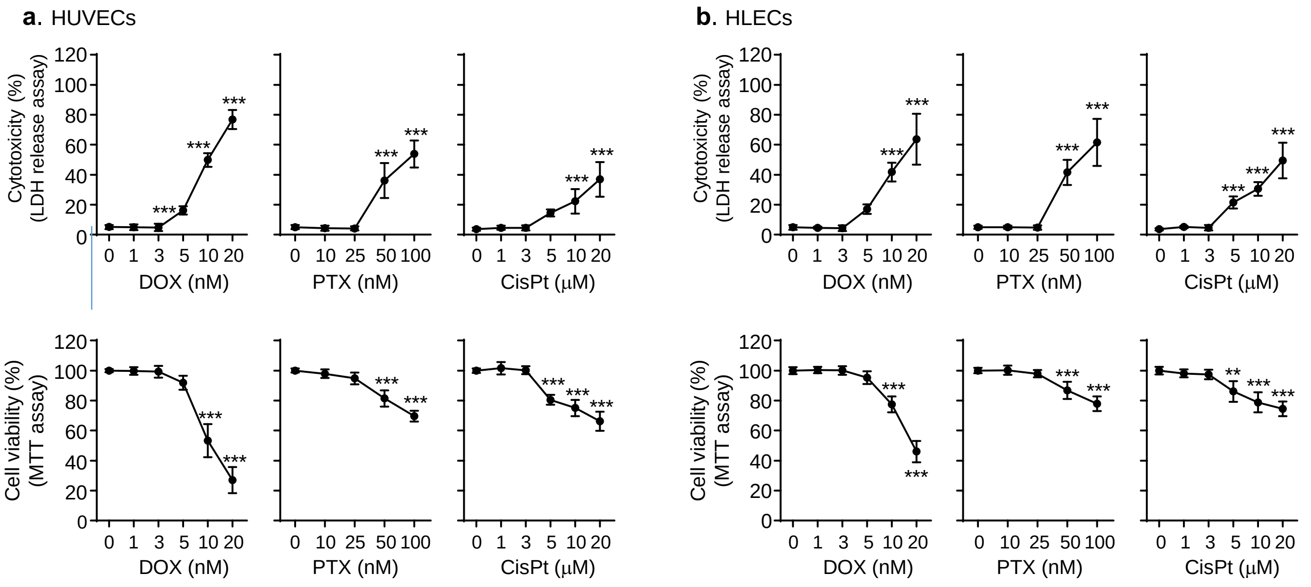

Supplemental Figure 1

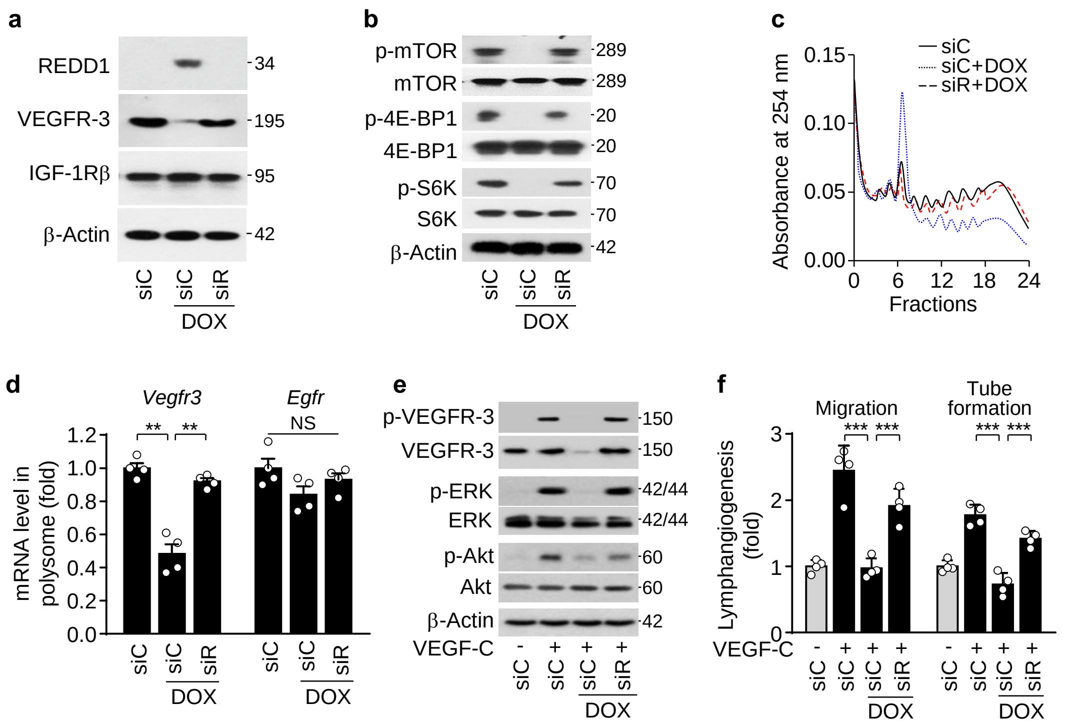

Supplemental Figure 2

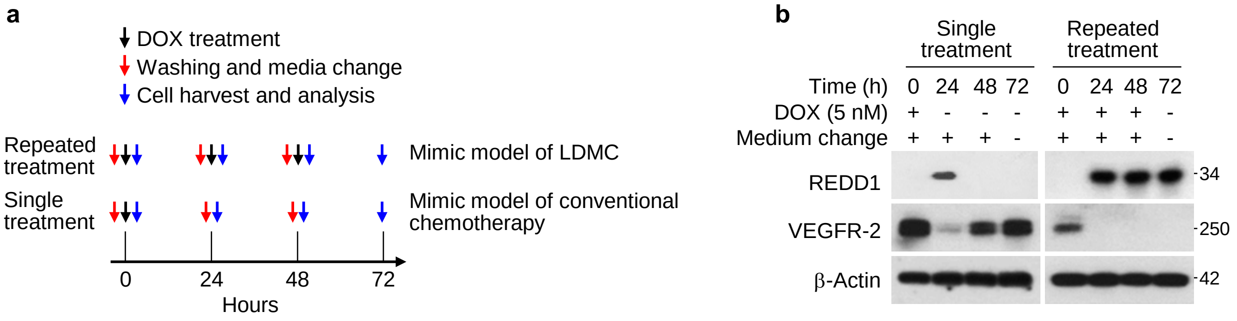

Supplemental Figure 3

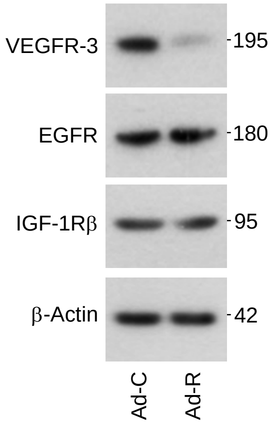

Supplemental Figure 4

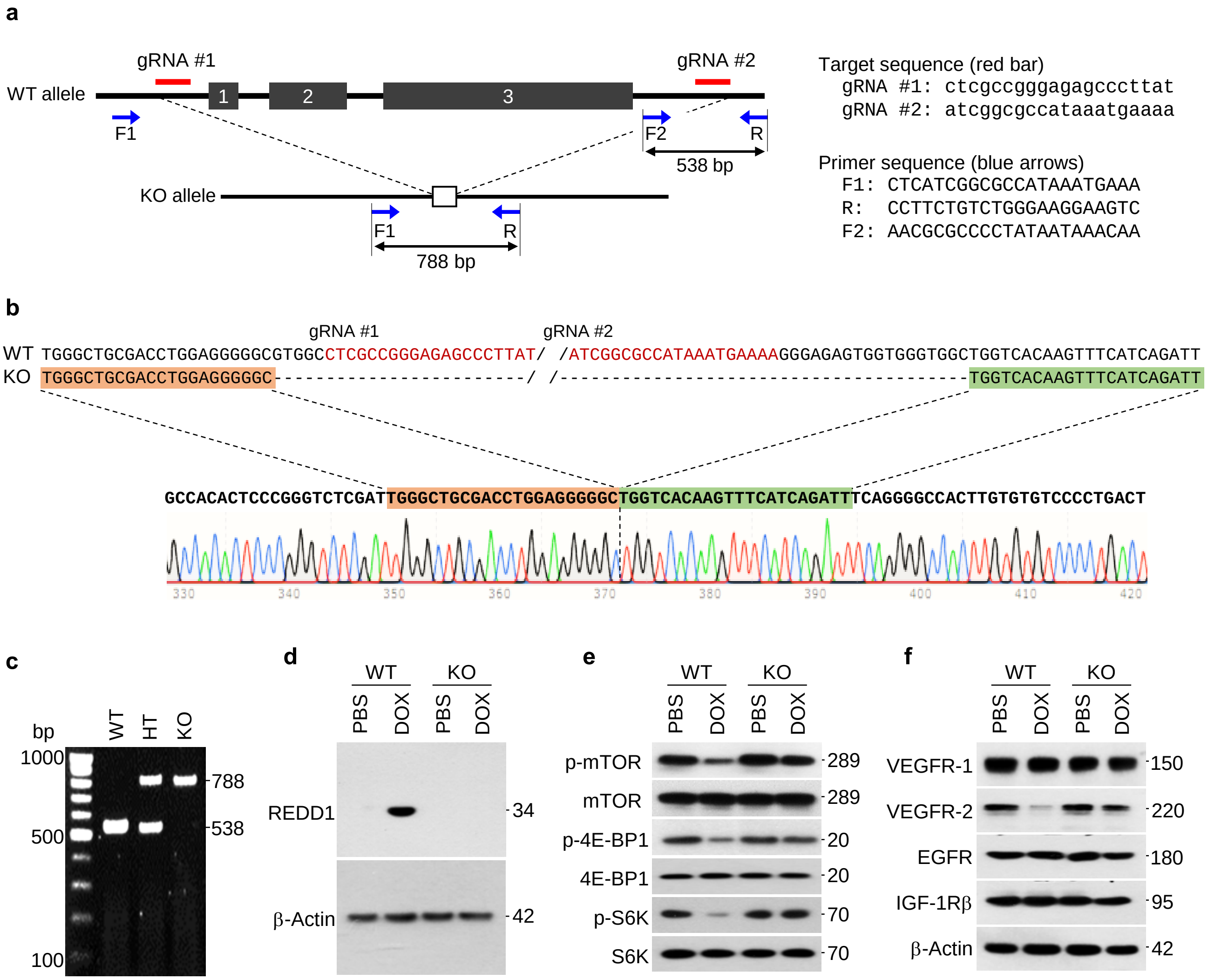

Supplemental Figure 5

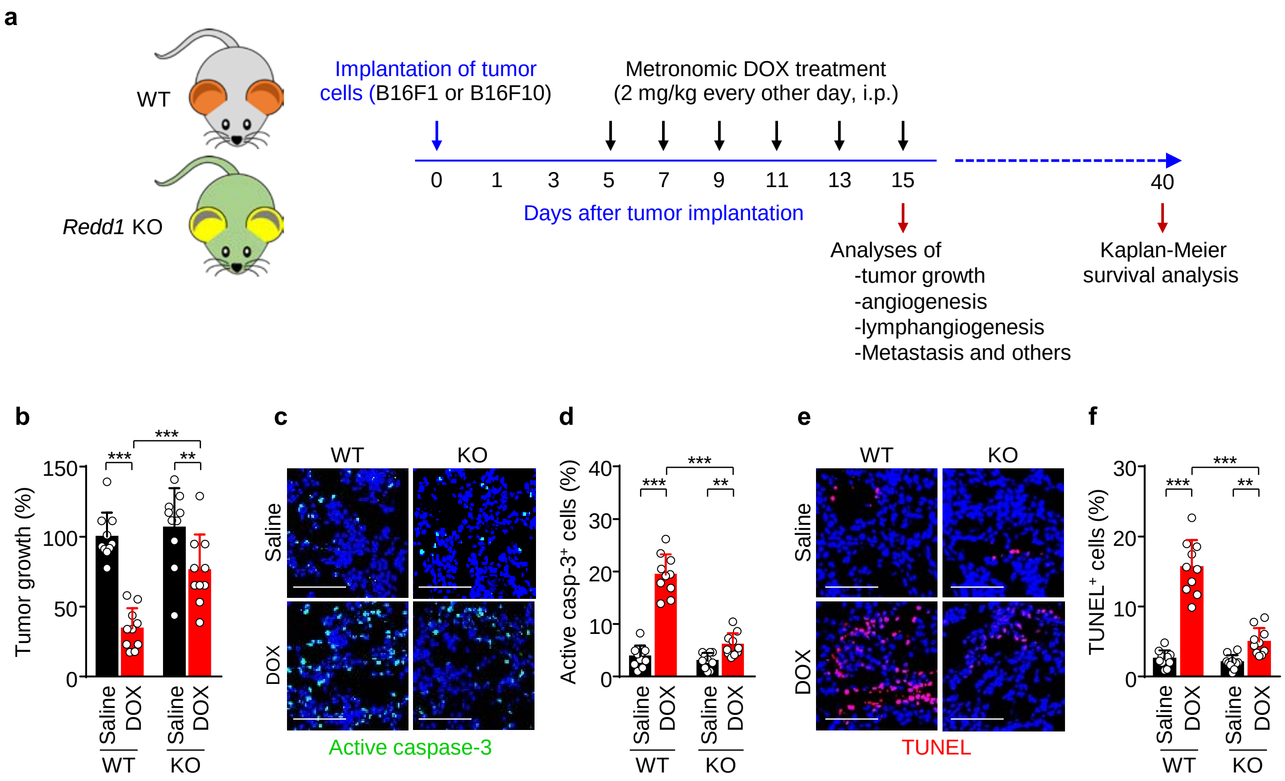

Supplemental Figure 6

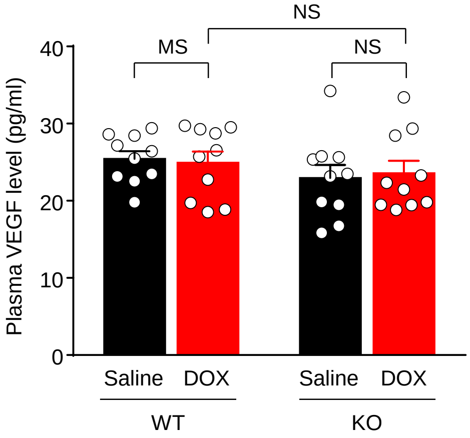

Supplemental Figure 7

Supplementary Figure Legends

Supplementary Figure 1. Dose-dependent cytotoxicity of chemotherapeutic drugs in endothelial cells. (**a**) HUVECs were treated with the indicated concentrations of doxorubicin (DOX), paclitaxel (PTX), and cisplatin (CisPt) for 24 h. Cytotoxicity and cell viability were determined via lactate dehydrogenase and MTT assays, respectively (n = 5). (**b**) HLECs were treated with the indicated concentrations of DOX, PTX, and CisPt for 24 h. Cytotoxicity and cell viability were analyzed (n = 5). Data are presented as the mean ± SD. ***P < 0.001.

Supplementary Figure 2. Low-dose DOX impairs *Vegfr-3* translation and lymphangiogenesis via REDD1 induction. *Vegfr-3* and *Egfr* mRNA translation and VEGF-C-induced lymphangiogenesis were examined in HLECs treated with DOX following transfection with control (siC) or *Redd1* siRNA (siR). (**a**, **b**) Western blotting for VEGFR-3 and IGF-1Rβ (**a**) as well as phosphorylated mTOR, 4E-BP1, and S6K (**b**). (**c**) Polysome profiling using sucrose density gradient ultracentrifugation. (**d**) qRT-PCR of *Vegfr-3* and *Egfr* mRNA associated with high-molecular-weight polysome (n = 4). (**e**) Phosphorylation of VEGFR-3, ERK, and Akt by VEGF-C was determined via western blotting. (**f**) Migration and tube formation of HLECs in response to VEGF-C were determined using Boyden chamber and Matrigel-based morphogenesis assays, respectively. (n = 4). Data are presented as the mean ± SD. **P < 0.01, ***P < 0.001; NS, not significant.

Supplementary Figure 3. Expression of REDD1 and VEGFR-2 in HUVECs treated with single or repeated low-dose DOX regimens. (**a**) Schematic illustrating the low-dose DOX regimen. (**b**) Western blotting of REDD1 and VEGFR-2.

Supplementary Figure 4. REDD1 overexpression downregulates VEGFR-3 but not EGFR and IGF-1Rβ expression in HLECs. HLECs were infected with control adenovirus (Ad-C) or Ad-*Redd1* (Ad-R). VEGFR-3, EGFR, and IGF-1Rβ levels were determined by western blotting.

Supplementary Figure 5. Generation of *Redd1*$^{-/-}$ mouse and functional analysis of its endothelial cells in response to DOX. (**a**) Schematic illustration of *Redd1* deletion using CRISPR/Casp9-based gene-editing system. The black boxes represent exons. Sequences of guide RNA (gRNA) and PCR primers are provided. (**b**) Genomic DNA sequence chromatogram of *Redd1*$^{-/-}$ mouse encompassing the deletion are shown. (**c**) PCR-based genotyping of mice carrying wild-type (WT), heterozygous mutant (HT, *Redd1*$^{+/-}$), or homozygous mutant *Redd1* (KO, *Redd1*$^{-/-}$). (**d**) Western blotting of REDD1 in WT and *Redd1*$^{-/-}$ mouse-derived lung endothelial cells treated with DOX. (**e**, **f**) Western blot analysis of the mTOR pathway (**e**) and VEGFR-1/2, EGFR, and IGF-1Rβ expression (**f**) in WT and *Redd1*$^{-/-}$ mouse-derived lung endothelial cells exposed to DOX.

Supplementary Figure 6. LDMC with DOX suppresses tumor growth via caspase-3 activation and apoptosis in WT but not *Redd1*$^{-/-}$ mice. (**a**) Diagram illustrating two syngeneic mouse models of low and high monastic melanomas (B16F1 and B16F10) and the treatment schedule of metronomic DOX. (**b**) Relative effects of LDMC with DOX on tumor volume at the endpoint in B16F1 tumor-bearing WT and *Redd1*$^{-/-}$ mice. (**c**) Representative images of caspase-3 staining in tumor sections. Scale bar, 100 μm. (**d**) Quantification of active caspase-3^{+} cells per HPF. (**e**) Representative images of TUNEL-positive tumor sections. Scale bar, 100 μm. (**f**) Quantification of TUNEL^{+} cells per HPF. Data are presented as the mean ±

SD (n = 10). **P < 0.01, ***P < 0.001.

Supplementary Figure 7. LDMC with DOX does not alter serum VEGF levels in B16F1 tumor-bearing mice. Tumor-bearing mice were treated with saline or DOX every other day for 15 days as shown in Supplementary Fig. 6A. VEGF serum levels were determined using an ELISA kit (MMV00; R&D Systems). Data are presented as the mean ± SD (n = 10). NS, not significant.
